# Supplementary material for: The causal role of circulating inflammatory markers in osteoporosis: a bidirectional Mendelian randomized study
Source: Front Immunol. 2024 Jul 18;15:1412298. doi: 10.3389/fimmu.2024.1412298 (PMC11291241; doi:10.3389/fimmu.2024.1412298)
Supplement: Supplementary file 1 [file DataSheet_1.zip › supplementary material/CIM-OSTEOPOROSIS /GCST90274805.csv_forest.pdf]

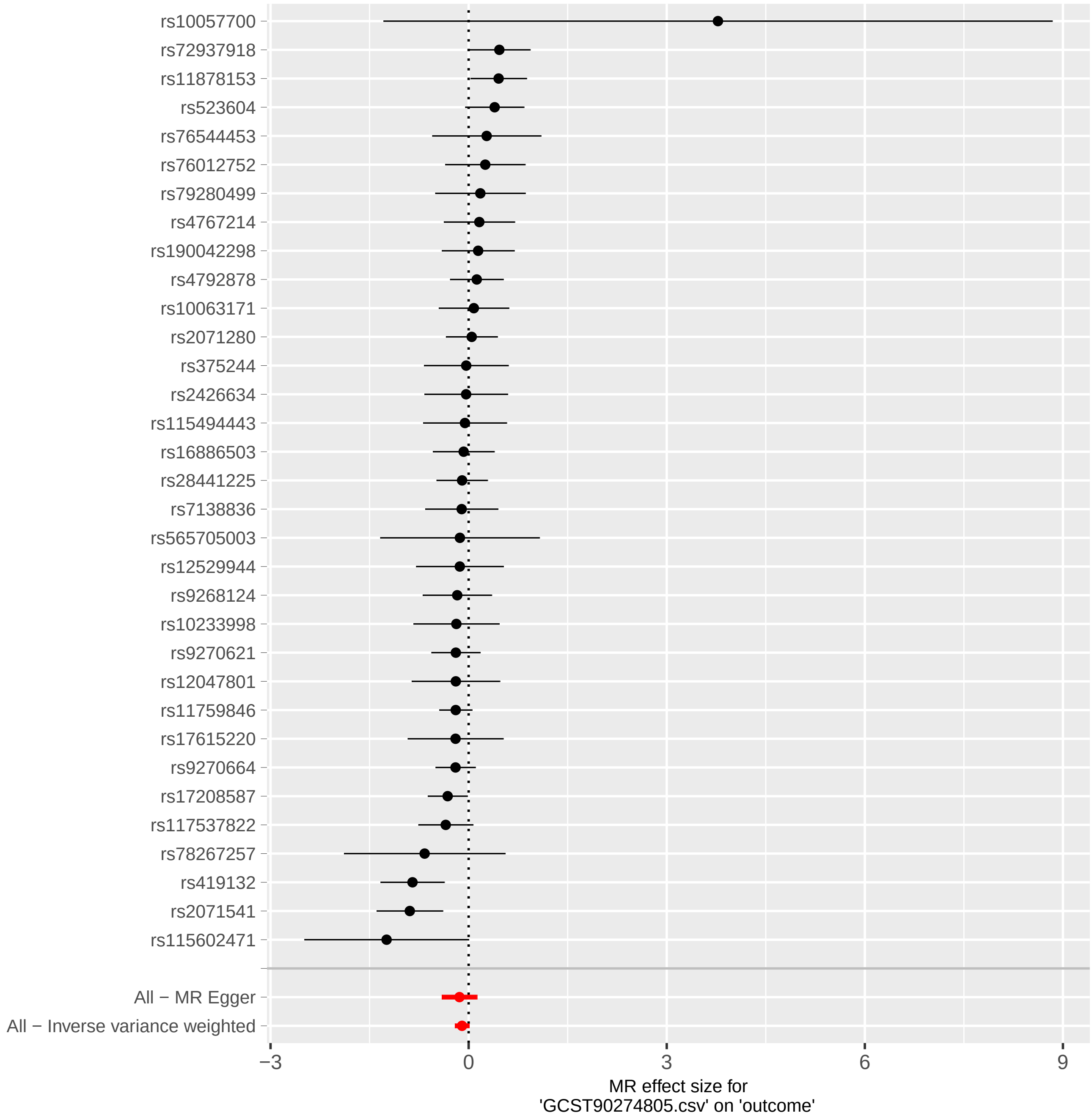
rs10057700
rs72937918
rs11878153
rs523604
rs76544453
rs76012752
rs79280499
rs4767214
rs190042298
rs4792878
rs10063171
rs2071280
rs375244
rs2426634
rs115494443
rs16886503
rs28441225
rs7138836
rs565705003
rs12529944
rs9268124
rs10233998
rs9270621
rs12047801
rs11759846
rs17615220
rs9270664
rs17208587
rs117537822
rs78267257
rs419132
rs2071541
rs115602471
All − MR Egger
All − Inverse variance weighted
−3
0
3
6
9
MR effect size for
'GCST90274805.csv' on 'outcome'
